# Flashes of UV-C light: An innovative method for stimulating plant defences

**DOI:** 10.1371/journal.pone.0235918

**Published:** 2020-07-09

**Authors:** Jawad Aarrouf, Laurent Urban

**Affiliations:** UMR Qualisud, Avignon Université, Avignon, France; National Research Council of Italy, ITALY

## Abstract

Leaves of lettuce, pepper, tomato and grapevine plants grown in greenhouse conditions were exposed to UV-C light for either 60 s or 1 s, using a specific LEDs-based device, and wavelengths and energy were the same among different light treatments. Doses of UV-C light that both effectively stimulated plant defences and were innocuous were determined beforehand. Tomato plants and lettuce plants were inoculated with *Botrytis cinerea*, pepper plants with *Phytophthora capsici*, and grapevine with *Plasmopara viticola*. In some experiments we investigated the effect of a repetition of treatments over periods of several days. All plants were inoculated 48 h after exposure to the last UV-C treatment. Lesions on surfaces were measured up to 12 days after inoculation, depending on the experiment and the pathogen. The results confirmed that UV-C light stimulates plant resistance; they show that irradiation for one second is more effective than irradiation for 60 s, and that repetition of treatments is more effective than single light treatments. Moreover a systemic effect was observed in unexposed leaves that were close to exposed leaves. The mechanisms of perception and of the signalling and metabolic pathways triggered by flashes of UV-C light vs. 60 s irradiation exposures are briefly discussed, as well as the prospects for field use of UV-C flashes in viticulture and horticulture.

## Introduction

There is the need to develop alternative or complementary solutions to pesticides (to decrease their use) that are effective, safe and economically viable. Chemical elicitors of plant defences are on the rise but their development is limited by inconsistent efficacy as a consequence of problems of formulation and stability in field conditions [1].

Physical elicitors do not present these drawbacks and have the additional advantage that they can be easily combined with other existing methods of treatment, either chemical or biological [2]. UV-B radiation has been observed to increase plant resistance to pathogens [3, 4]. UV-B light is known to act through signalling pathways, involving notably mitogen-activated protein kinases, which closely resemble those for pathogens [5]. It has also been observed that UV-C light can stimulate plant and crop defences against *Botrytis cinerea* and *Sclerotinia minor* [6–11]. UV-B light requires extensive periods of irradiation (several hours or days) to be effective. This limits its use in greenhouse conditions, where lamps are in stationary positions, whereas effective doses of UV-C light can be supplied to plants and crops typically in 60 s [2, 12]. However actual use of UV-C light in field conditions will require the capacity to deliver effective doses in much less time than that. Therefore there is a need to design lamps that are able to deliver effective doses that are ideally one second or less. It also needs to be determined whether doses delivered in such a short time still are able to stimulate plant defences.

We tested in this trial the hypothesis that UV-C light in flashes of 1 s are capable of stimulating plant defences against several fungal and oomycete diseases. We moreover tested the idea that flashes of 1 s are more efficient than irradiations for 60 s by using an original device specifically designed for this purpose, i.e. maintaining equal wavelengths and energy among light treatments in a range of exposures from 1 to 60 s. Eventually we evaluated the effects of repetition of light treatments and systemic effects.

## Materials and methods

### LEDs-based lamp

**Fig 1:**
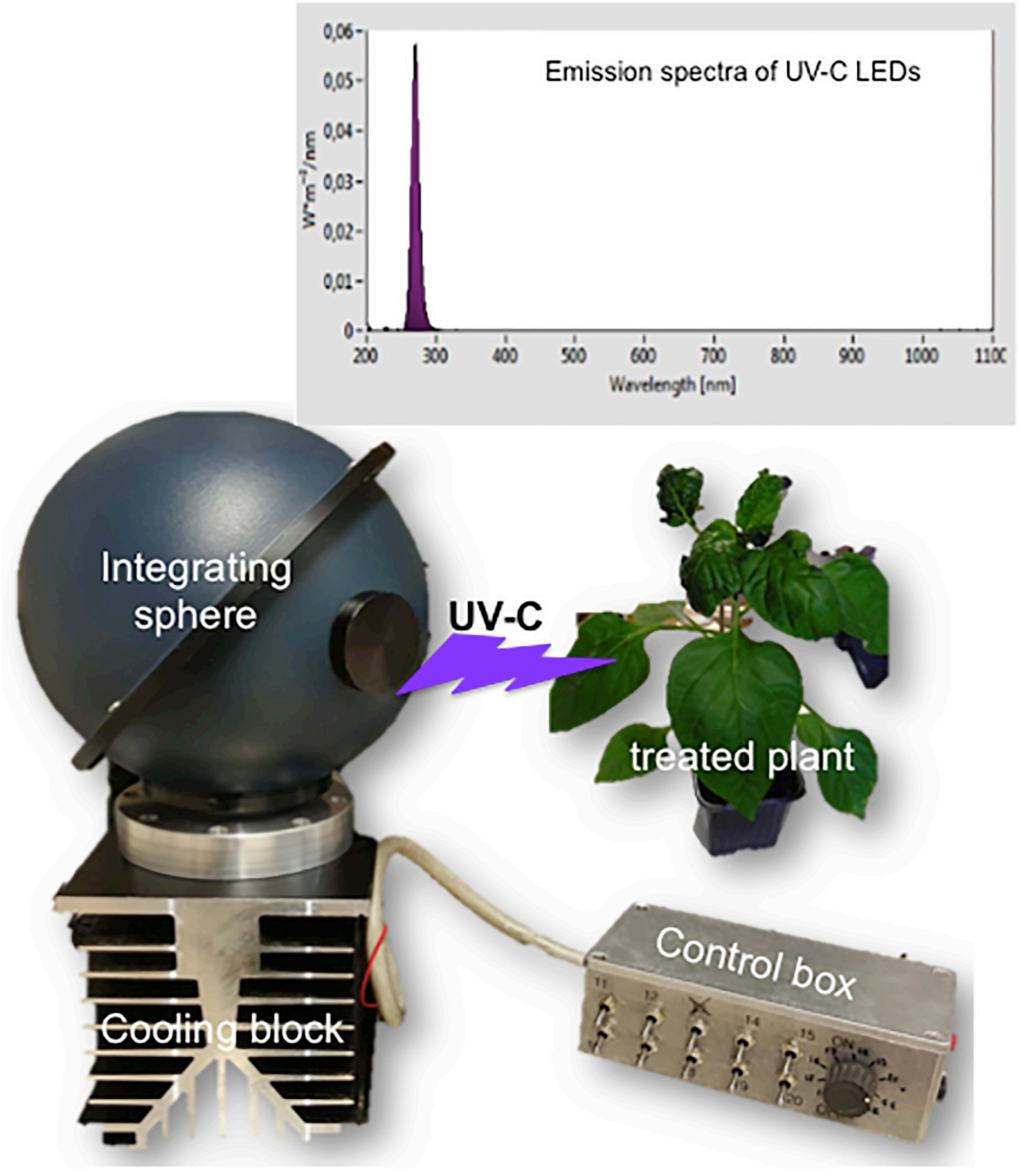
Photo of the device used for supplying UV-C light to plants. The inside of the integrating sphere accommodates 15 UV-C LEDs on a printed circuit board. The cap of the 5 cm^2^ window is removed and the window placed directly on the leaves for treatments. A typical light spectrum is shown.

The lamp used for the trials was made of 15 light emitting diodes (LEDs) on a printed circuit board fitted inside an integrating sphere (Labsphere Inc., North Sutton, NH, USA) (Fig 1). The LEDs consisted of SMD LEDs (Crystal IS Inc., Green Island, NY, USA). These LEDs are made of an alloy between gallium nitride (3.6 eV) and aluminium nitride (6.2 eV) and generate photons at 265 nm (Fig 1). They are capable of delivering more than 20 mW each in the pulse mode, depending on the temperature. A specific power supply and Peltier cooling systems were designed to maximize the light output of the LEDs. Calculation and direct measurement (Hera spectrophotometer, Pro-Lite, Marcillac, France) showed that it was possible to reach 100 mW cm^-2^ (1 kW m^-2^, corresponding to 2214 μmol photons m^-2^ s^-1^) at the level of the 5 cm^2^ window at the bottom of the integrating sphere [13].

### Plant culture

**Fig 2:**
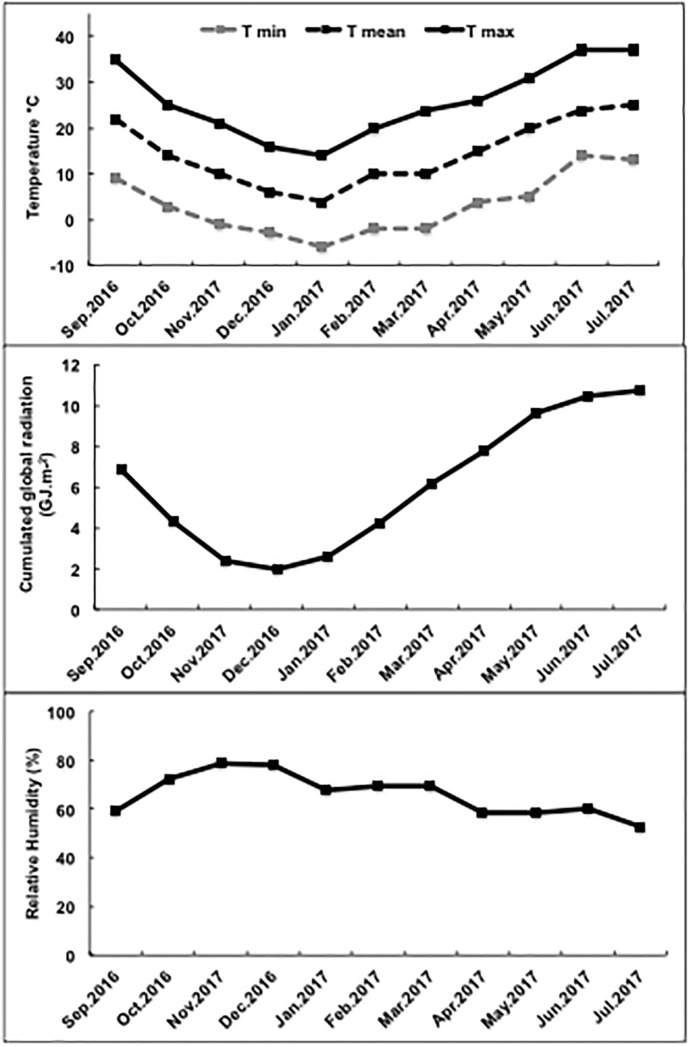
Daily mean, maximum and minimum temperatures and daily cumulated global solar radiation, and daily mean relative humidity from September 2016 to July 2017.

Experiments were conducted in the Avignon University greenhouses, between October 2016 and May 2017 (Table 1). Daily mean maximum and minimum temperatures and relative humidities, and daily cumulated global solar radiation were recorded (Fig 2). Lettuce, tomato and pepper seeds were sown in 1 cm^3^ rockwool cubes in a glasshouse at 25 ± 2°C. One week after sowing, the cubes, each containing one plantlet, were transferred into plastic pots (5 L), filled with a commercial growing medium (Klasmann Deilmann Gmbh, Bremen, Germany) containing 80% organic matter (pH = 6). The plants were then grown for 4 weeks at 24/16 °C (day/night temperatures) at ambient CO_2_. For grapevines, rooted cuttings, cv. Cabernet Sauvignon, were cultivated in plastic pots (10 L), filled with a commercial growing medium (Platinium, Avignon, France) containing 85% organic matter (pH = 6.5). Plants were grown for 10 weeks under controlled conditions at 25/20 °C (day/night temperatures) at ambient CO_2_. We used fertilizers with the following compositions: 5% N, 5% P_2_O_5_, 7% K_2_O, 2.5% MgO, 12% SO_3_ and 13% CaO (lettuce, 30 g per plant); 5% N, 5% P_2_O_5_, 8% K_2_O, 3% MgO, 11% SO_3_ and 14% CaO (tomatoes, 50 g per plant); 6% N, 3% P_2_O_5_, 3% K_2_O, 6% MgO, 2% SO_3_ and 14.6% CaO (peppers, 30 g per plant); 7% N, 4% P_2_O_5_, 7% K_2_O, and 6% MgO (grapevines, 60 g per plant). A regular water regime was applied for all of the plants every two days. No pesticides were applied during the whole period of the trials. Control and treated plants were randomly distributed on part of a bench that was selected for being homogeneous in terms of light and temperature.

**Table 1:** Cultivation periods of the different species used in the experiments.

	Experiment 1	Experiment 2	Experiment 3
**Tomato**	03 Oct—07 Nov	19 Dec—23 Jan	6 Mar—10 Apr
**Lettuce**	17 Oct—21 Nov	09 Jan—13 Feb	20 Mar—24 Apr
**Pepper**	31 Oct—05 Dec	23 Jan—27 Feb	28 Mar—2 May
**Grapevine**	-	26 Dec—06 Mar	20 Mar—8 May

### UV-C treatments

Three experiments were performed (Table 1). There were five plants per UV-C light treatment and five plants served as untreated controls. Three tagged leaves per plant, of similar age and exposure to light, were submitted to UV-C treatments or used as controls (*n* = 15). UV-C treatments consisted in exposing one single 5 cm^2^ spot per leaf for either 1 or 60 s. Beforehand we determined the doses of UV-C light that are effective for stimulating plant defences without any negative effects on plants. The dose of UV-C light used in this study is the highest dose that can be delivered in 1 s by the LEDs system described above, i.e.1 kJ m^-2^. The absence of negative effects on plants was assessed visually and by measurements of chlorophyll fluorescence, ChlF.

### Chlorophyll fluorescence measurements

ChlF measurements were performed on treated and control leaves, ca. 48 hours after UV-C treatments, around 2 pm, with a Pocket PEA chlorophyll fluorimeter (Hansatech Instruments, King’s Lynn, Norfolk, United Kingdom). Leaves were dark-adapted for 1200 s with a lightweight plastic leaf clip prior to measurements. The transients were induced by 1 s illumination with a single light-emitting diode providing a fully saturating photon flux density of 3500 μmol photons m^-2^ s^-1^ with a peak wavelength of 627 nm at the sample surface, and homogeneous irradiation. The ChlF intensity at 50 μs was considered as F_0_ [14]. Several parameters derived from measurements of induction curves of maximal ChlF were calculated, the ratio of variable ChlF (F_v_) to maximum ChlF (F_m_), F_v_/F_m_, and the Performance Index (PI_abs_), a plant vitality indicator [15], which is generally believed to be a more sensitive parameter than F_v_/F_m_ even though there are contradictory observations [16]. We also calculated V_k_/V_j_, which represents the ratio of variable ChlF at 300 μs (K-step) to variable ChlF at 2 ms (J-step), and S_m_, the normalized area above the ChlF induction curve.

#### Experiment 1: The effect of single UV-C flashes

15 lettuce plants, 15 tomato plants and 15 pepper plants were used in this experiment between October and December 2016 (Table 1). After four weeks of cultivation, leaf spots of 5 cm^2^ were treated by single exposures to UV-C light at 1 kJ m^-2^ supplied over 1 s (1 kW m^-2^) or at the same cumulative dose supplied over 60 s. Two days later, leaves were detached and placed separately in plastic Petri dishes on moistened filter paper for inoculation.

#### Experiment 2: The effect of repeated UV-C flashes

15 lettuce plants, 15 tomato plants, 15 pepper plants and 15 grapevine plants were used between December 2016 and March 2017 (Table 1). After four weeks of culture, leaf spots of 5 cm^2^ were exposed to UV-C light at 1 kJ m^-2^ for 1 s (1 kW m^-2^) in one treatment. UV-C flashes were then repeated three times on different spots of the same leaves, at 48 h intervals. In another treatment, lettuce and tomato leaves were exposed only to a single flash of UV-C light, supplied the same day as the day of the last exposure to UV-C light in the previous treatment. Two days later, leaves were detached and placed separately in plastic Petri dishes on moistened filter paper for inoculation.

#### Experiment 3: Systemic effects of UV-C flashes

Ten lettuce plants, ten tomato plants, ten pepper plants and ten grapevine plants were used in this experiment between March and May 2017 (Table 1). After either four weeks of culture (lettuce, tomatoes, peppers) or ten weeks (grapevines), leaf spots of 5 cm^2^ were exposed to UV-C light at 1 kJ m^-2^ for 1 s (1 kW m^-2^). UV-C flashes were repeated three times on different spots of the same leaves, at 48 h intervals. Two days after the last UV-C treatment, the 15 tagged leaves exposed to flashes of UV-C light were detached. 15 untreated leaves of similar age were also taken randomly among the treated plants, and 15 leaves from the five control plants. Leaves were placed separately in plastic Petri dishes on moistened filter paper for inoculation.

### Pathogen culture, inoculation and analysis of necrotic spots

*Botrytis cinerea* inoculum was produced in three days on a medium made of potato dextrose agar (39 g L^−1^ Difco, Detroit, USA), in a growth chamber at 21 °C, with a 14 h/10 h photoperiod. *Phytophthora capsici* mycelium was grown over 8 days on V8 juice agar medium in a growth chamber at 22°C, with a 12 h/12 h photoperiod. *Plasmopara viticola* inoculum was produced from sporangia derived from a susceptible cultivar of *Vitis vinifera*. The leaves were maintained under moist conditions overnight to induce maximal sporulation. Sporangia were recovered by soaking infected leaves in cold (4 °C) distilled water, and the dilution was adjusted to reach a concentration of 5 × 10^5^ sporangia.ml^−1^ using a Malassez haemocytometer.

**Fig 3:**
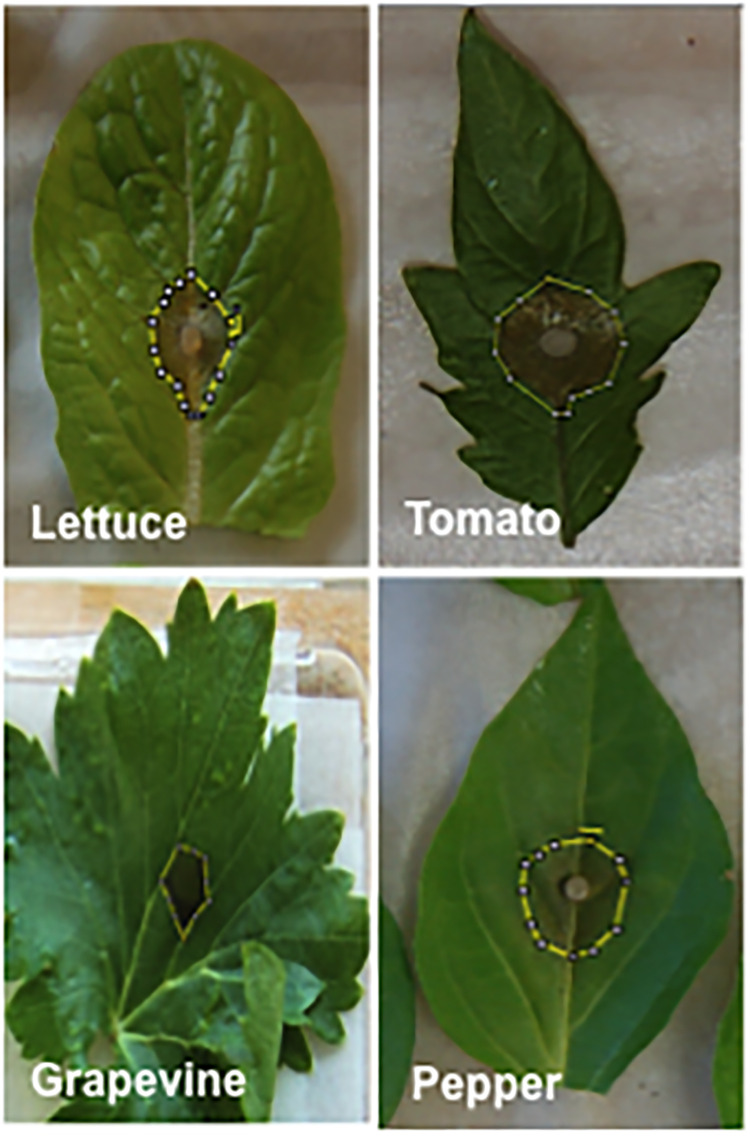
Position of mycelium plugs and typical lesion areas used for image analysis.

Leaves were inoculated by depositing either a mycelium plug of *Botrytis cinerea* on the middle of the leaf (lettuce and tomato), or a mycelium plug of *Phytophthora capsici* (pepper) (Fig 3). Grapevine leaves were inoculated by depositing a drop of 20 μl suspension of sporangia of *Plasmopara viticola* on the middle of the leaf (Fig 3).

The leaves were photographed from two to 12 days after inoculation, depending on the species, and the lesion areas were assessed with an image analysis software (ImageJ, US National Institutes of Health, Bethesda, MD, USA), as shown in Fig 3.

### Statistical analysis

For each experiment, the Kruskal-Wallis non-parametric statistical test was applied (*n* = 15). The data were expressed as the means ± standard error, and statistical significance was set at P < 0.05. All statistical analyses were performed using XLSTAT software (Addinsoft, Deutschland, Andernach, Germany).

## Results

**Fig 4:**
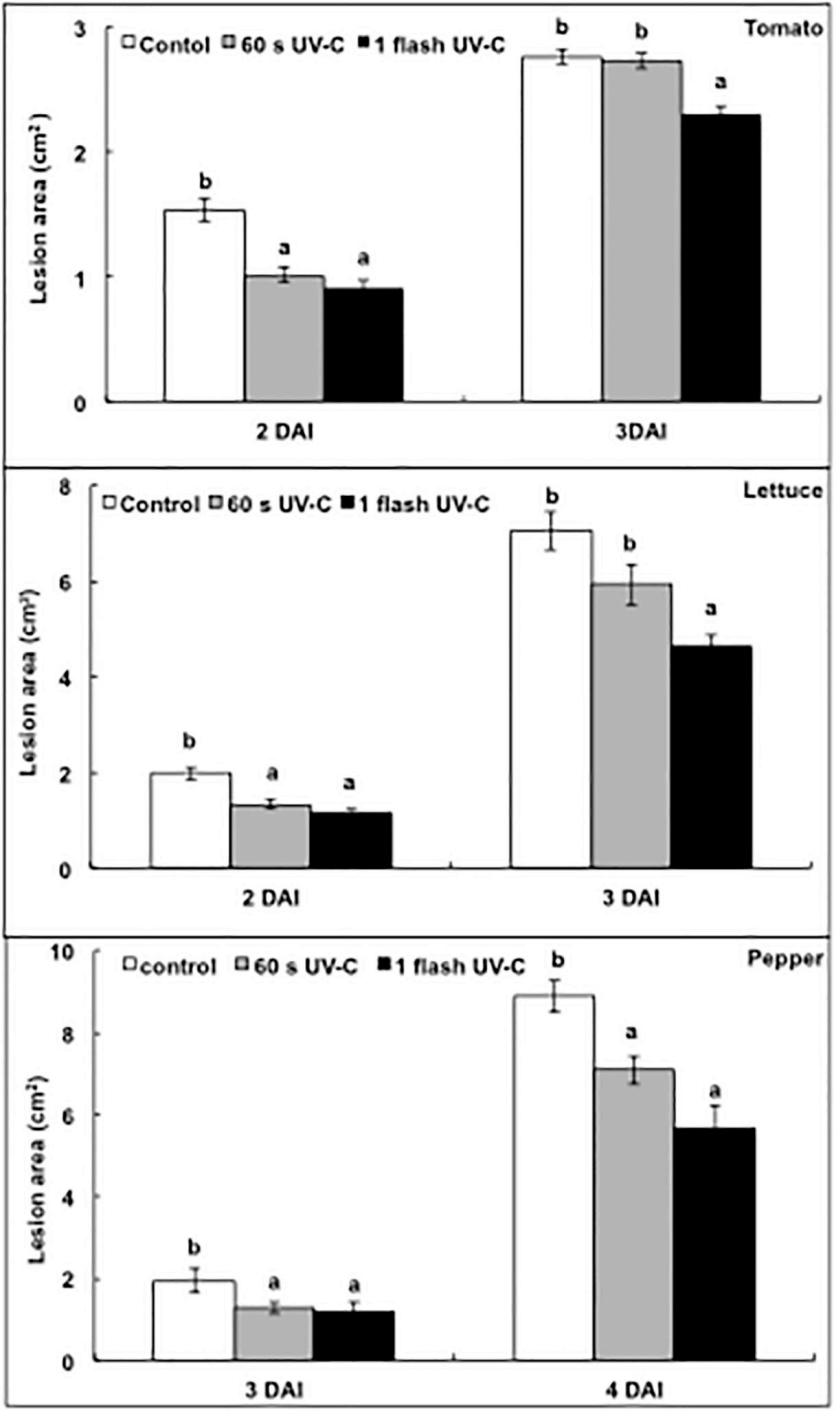
Effect of UV-C light at 1 or 60 s irradiations on plant defences. Leaves were exposed to a single dose of UV-C light of 1 kJ m^−2^, for either 1 or 60 s. Two days after UV-C treatments, tomato and lettuce leaves were inoculated with *Botrytis cinerea*, and pepper leaves were inoculated with *Phytophthora capsici*. Lesion areas (cm^2^) of tomato (A), lettuce (B) and pepper (C) leaves were measured. DAI stands for days after inoculation. The bars represent standard errors (*n* = 15). Different letters indicate significant differences according to the Kruskal-Wallis test (P < 0.05).

Lesions areas increased over time in all control and treated plants, but single 60 s irradiations by UV-C light reduced them by 35% in tomatoes two days after inoculation with *Botrytis cinerea*, by 17% in lettuce three days after inoculation with *Botrytis cinerea*, and by 35% and 21% in peppers, three and four days, respectively, after inoculation with *Phytophthora capsici* (Fig 4). There was no significant or substantial reduction in lesion areas in tomatoes and in lettuce three days after inoculation. Single flashes of UV-C light reduced lesion areas by 41% and 17% in tomato, two and three days, respectively, after inoculation with *Botrytis cinerea*, by 42% and 35% in lettuce, two and three days, respectively, after inoculation by *Botrytis cinerea*, and by 39% and 37% in peppers, three and four days, respectively, after inoculation by *Phytophthora capsici* (Fig 4). For the three pathosystems, single flashes of UV-C light had a more pronounced effect than a single 60 s irradiation (Fig 4).

PI_abs_ did not decrease as a consequence of UV-C light treatments (Table 2). PI_abs_ in the 60 s treatment in tomatoes and in the 1 s treatment in lettuce and grapevines was even higher than in the control. Similarly, F_v_/F_m_ did not decrease as a consequence of UV-C light treatments (Table 2). On the contrary, F_v_/F_m_ was higher than in the control in the 60 s treatment in peppers, and in the 1 s treatment in lettuce, tomatoes and grapevines. F_0_ was lower in the 1 s treatment in lettuce and grapevines than in the control, whereas S_m_ was higher in the 1 s and the 60 s treatments in lettuce and grapevines than in the control. V_k_/V_j_ was not affected by any of the treatments.

**Table 2:** Effect of UV-C irradiation on the Performance Index of Strasser [15] and on the parameters derived from induction curves of maximal ChlF that have been proposed to be damage indicators, including F_v_/F_m_ [16]. Data represent means + SE. *n* = 18. Different letters for a given species and a given parameter correspond to differences significant at the 5% threshold.

	PI_abs_	F_v_/F_m_	F_0_	S_m_	V_k_/V_j_
**Lettuce control**	2.59 ± 0.18 *a*	0.783 ± 0.004 *a*	5935 ± 158 *b*	23.3 ± 0.4 *a*	1518 ± 52 *a*
**Lettuce 1 s**	2.91 + 0.17 *b*	0.803 ± 0.003 *b*	5361 ± 71 *a*	24.9 ± 0.5 *b*	1473 ± 34 *a*
**Lettuce 60 s**	2.70 + 0.13 *ab*	0.793 ± 0.004 *ab*	5696 ± 100 *b*	25.9 ± 0.4 *b*	1470 ± 44 *a*
**Tomato control**	3.45 ± 0.40 *a*	0.803 ± 0.003 *a*	4798 ± 117 *a*	13.2 ± 0.3 *a*	1658 ± 51 *a*
**Tomato 1 s**	5.04 ± 0.74 *ab*	0.813 ± 0.004 *b*	5138 ± 161 *a*	13.9 ± 0.5 *a*	1775 ± 85 *a*
**Tomato 60 s**	5.53 ± 0.73 *b*	0.812 ± 0.003 *ab*	4789 ± 214 *a*	13.9 ± 0.5 *a*	1656 ± 108
**Pepper control**	3.20 ± 0.33 *a*	0.787 ± 0.004 *a*	6390 ± 144 *a*	16.9 ± 0.6 *a*	1786 ± 51 *a*
**Pepper 1 s**	2.59 ± 0.23 *a*	0.794 ± 0.004 *ab*	6289 ± 192 *a*	16.6 ± 0.5 *a*	1718 ± 59 *a*
**Pepper 60 s**	3.00 ± 0.15 *a*	0.799 ± 0.002 *b*	6119 ± 207 *a*	16.7 ± 0.4 *a*	1744 ± 51 *a*
**Grapevine control**	2.59 ± 0.18 *a*	0.783 ± 0.004 *a*	5935 ± 157 *b*	23.3 ± 0.4 *a*	1518 + 51 *a*
**Grapevine 1 s**	2.90 ± 0.17 *b*	0.803 ± 0.003 *b*	5361 ± 70 *a*	24.8 ± 0.5 *b*	1472 + 34 *a*
**Grapevine 60 s**	2.67 ± 0.15 *ab*	0.793 ± 0.004 *ab*	5691 ± 100 *ab*	25.9 ± 0.4 *b*	1470 + 45 *a*

**Fig 5:**
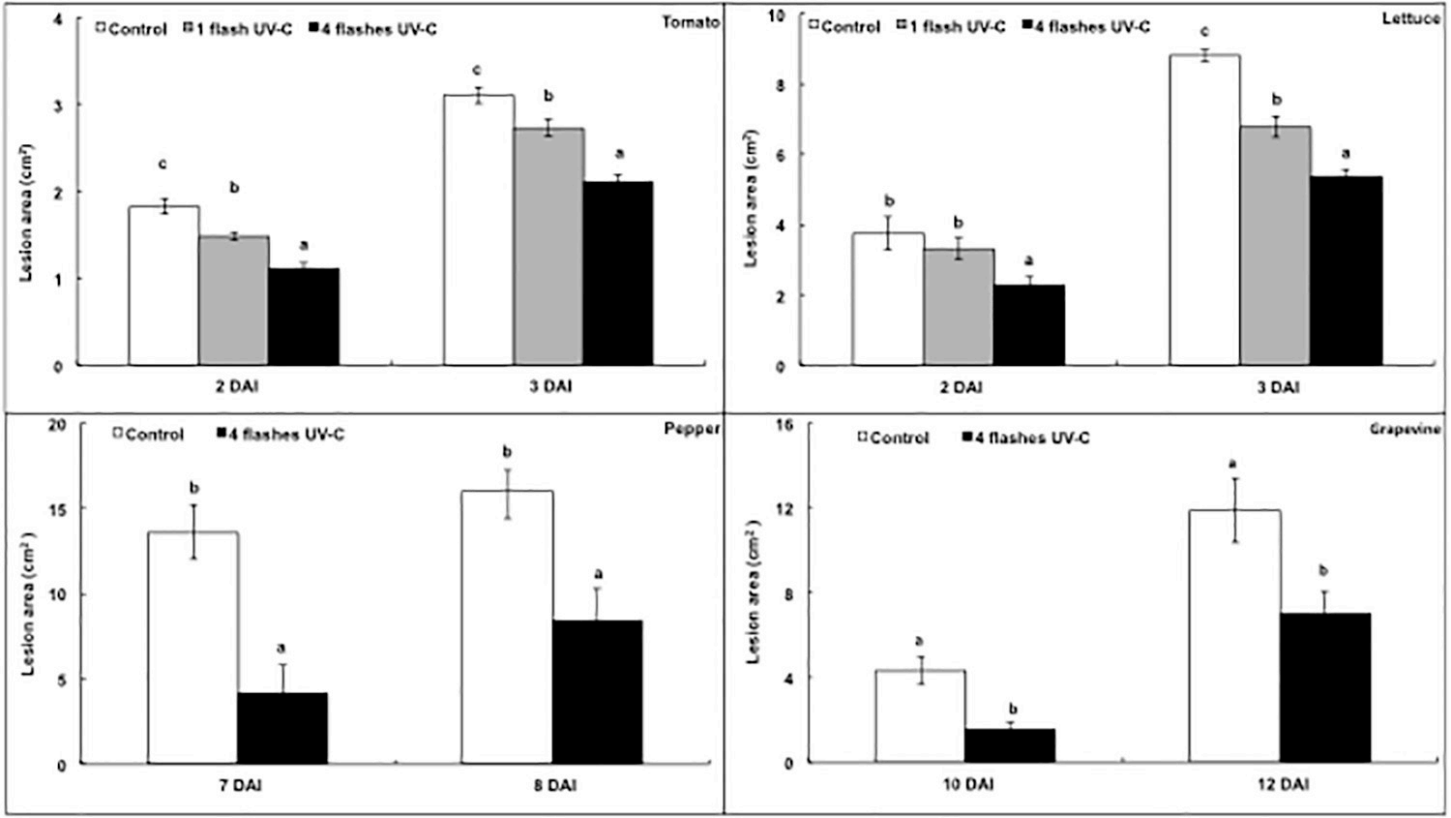
Effect of repeated flashes of UV-C light on plant defences. Tomato and lettuce leaves were exposed to flashes of UV-C light of 1 kW m^−2^ each, either one or four times. Pepper and grapevine leaves were exposed only to a dose of 1 kJ m^−2^, repeated four times. Two days after UV-C treatments, tomato and lettuce leaves were inoculated with *Botrytis cinerea*, pepper leaves with *Phytophthora capsici* and grapevine leaves with *Plasmopara viticola*. Lesion areas (cm^2^) of tomato (A), lettuce (B), pepper (C) and grapevine (D) leaves were measured. DAI stands for days after inoculation. The bars represent standard errors (*n* = 15). Different letters indicate significant differences according to the Kruskal-Wallis test (P < 0.05).

When 1 s UV-C light treatments were repeated four times, with 48 h between exposures, lesion areas were reduced in tomatoes by 40% and 33% two and three days, respectively, after inoculation with *Botrytis cinerea* (Fig 5). There was also an effect of single flashes of UV-C light but a less marked one, since lesion areas were only reduced by 19% and 13%, two and three days, respectively, after inoculation with *Botrytis cinerea* (Fig 5). In lettuce, lesion areas were reduced by single flashes of UV-C light by 24% three days after inoculation with *Botrytis cinerea* (Fig 5). For plants treated four times, the reduction of lesion area was already significant two days after inoculation when compared to either untreated control plants or single treated plants. It reached 39% when compared to untreated control plants three days after inoculation. Repeated treatments by UV-C light were also tested against *Phytophthora capsici* in peppers and against *Plasmopara viticola* in grapevines. They resulted in a 70% and 48% reduction in lesion areas 7 and 8 days, respectively, after inoculation of peppers with *Phytophthora capsici* (Fig 5). They also resulted in a 65% and 41% reduction in lesion areas 10 days and 12 days, respectively, after inoculation of grapevines with *Plasmopara viticola* (Fig 5). This confirmed the potential of repeated flashes of UV-C light to stimulate plant defences against several fungal plant diseases.

**Fig 6:**
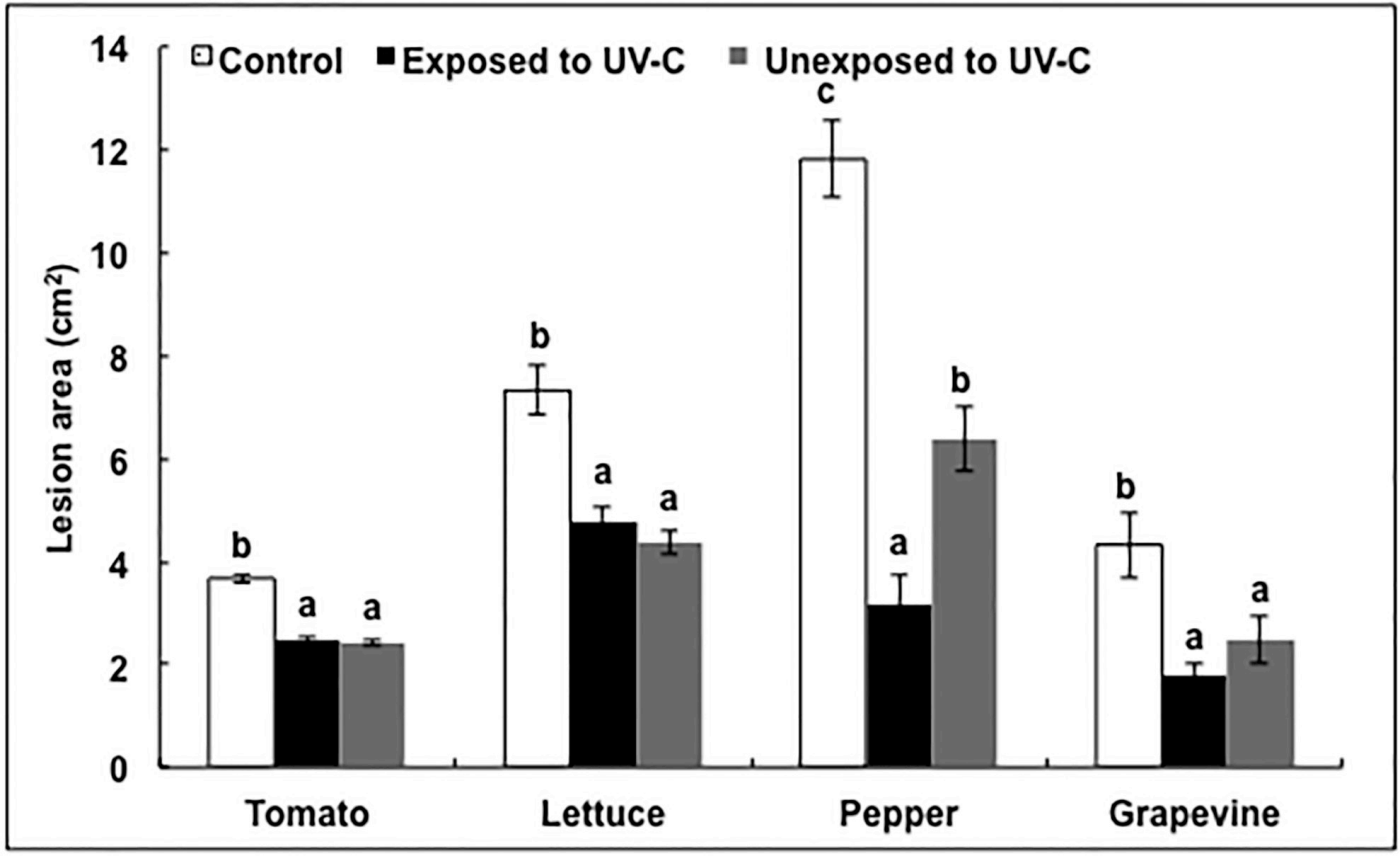
Effect of flashes of UV-C light repeated four times on defences of treated and of nearby untreated leaves. Tomato (A), lettuce (B), pepper (C) and grapevine (D) leaves were exposed to flashes of UV-C light of 1 kW m^−2^, repeated four times. Two days after UV-C treatments, tomato and lettuce leaves were inoculated with *Botrytis cinerea*, pepper leaves with *Phytophthora capsici* and grapevine leaves with *Plasmopara viticola*. Lesion areas were measured on exposed leaves and on nearby unexposed leaves, after three days for tomatoes, lettuce and peppers, and after 10 days for grapevines. The bars represent standard errors (*n* = 15). Different letters indicate significant differences according to the Kruskal-Wallis test (P < 0.05).

Lesion areas were not only reduced in leaves directly exposed to repeated UV-C light treatments, but also in nearby, non-exposed tomato, lettuce, pepper and grapevine leaves taken from the same treated plants (Fig 6). The effect was of similar magnitude in non-exposed tomato, lettuce and grapevine leaves as in the leaves exposed to UV-C treatments, but the effect was less pronounced in non-exposed pepper leaves than it was in the leaves exposed to UV-C treatments.

## Discussion

### UV-C light stimulates plant defences

Results obtained as part of this study confirm previous observations showing that UV-C light, either under the form of exposures of 60 s or of 1s (Fig 4), can stimulate plant defences [6–11]. See also Terry and Joyce for older references [17]. Not much is known about the mechanisms of perception and about the signalling and metabolic pathways triggered or stimulated by hormetic doses of UV-C light [2, 12]. It is reasonable to hypothesize that the high energy levels supplied by UV-C light are at the origin of the production of reactive oxygen species (ROS) by either the photosynthetic machinery in the chloroplasts, NADP(H) oxidase activity at the membrane level, xanthine oxidase activity in peroxisomes or NADP-malic enzyme activity in mitochondria [2]. Even though ROS are generally efficiently dealt with by the antioxidants, antioxidant enzymes and antioxidant systems existing in all compartments of the cell, they can be at the origin of oxidative signalling resulting in the triggering or the upregulation of signalling and metabolic pathways associated with the production of secondary defence compounds. In addition, direct lipid damage or peroxidation by ROS can be at the origin of linolenic acid oxidation products, which serve as precursors in the synthesis of jasmonic acid, an hormone playing important roles in plant responses to biotic stressors [18]. In addition to the ROS hypothesis, photoreceptors could be involved, notably phototropin [19] or UVR8 [20]. The latter protein has a demonstrated role in UV-B perception and an action spectrum overlapping largely over the UV-C domain [21]. In addition, UV-C light was recently found to alleviate transcriptional gene silencing in *Arabidopsis* [22], an indicator that UV-C light has epigenetic effects. We should certainly pay more attention to the latter in the future considering that there is growing evidence for the role played by epigenetic mechanisms in the control of plant immunity [23, 24].

### Flashes of UV-C light have a strong potential for stimulating plant defences

One second flashes of UV-C light stimulated plant defences at least as well, and in most cases even better than conventional irradiation exposures of 60 s (Fig 4). There is a strong consensus in the scientific community suggesting that doses (in J m^-2^) matter in the observed effects of UV light, not the time of exposure. In other words, it is believed that the response of plants does not change if they are exposed over a short or a lengthy period of time, as long as the dose considered to be effective is delivered. This idea led to the concept of maximal acceptable doses, alias MAD [25]. In plant studies, much like in human health research, authors have also considered that doses matter, not the time of exposure. It has been repeatedly stated that the efficiency of UV-C light is dose-dependent and that a longer duration of low radiance (W m-2) has the same effect as a short but strong irradiation [26–28].

Taking a view opposing that of the scientific community we observed that for a given effective dose of UV-C light, the efficiency is higher when UV-C light is supplied as a flash of one second, rather than under the form of an irradiation of low intensity delivered over an extensive period of time (of 60 s) (Fig 4). The superiority for plant resistance of very short versus long periods of irradiation by UV-C light is intriguing, and suggests that mechanisms of light perception, signalling and metabolic pathways may be different when UV-C light is supplied as flashes instead of prolonged irradiations.

### ChlF damage and performance indicators are not affected negatively by treatments

Substantially lower values of F_v_/F_m_ are indicators of photodamage [29]. Similarly, higher values of F_0_ suggest damage [30]. We did not observe any decrease in F_v_/F_m_ nor any increase in F_0_ as a consequence of UV-C light treatments (Table 2). Limitation/inactivation, possibly through damage to the oxygen-evolving complex (OEC), may be observed and assessed through the increase in V_k_/V_j_ [31, 32]. We did not observe any increase in V_k_/V_j_ either as a consequence of UV-C light treatments (Table 2). By contrast, a slight increase in S_m_ was observed in lettuce and grapevine as a consequence of the 1s and 60 s UV-C light treatments (Table 2). S_m_ is assumed to be proportional to the pool size of electron carriers, and decreases in S_m_ are suspected to be indicators of stress-associated damage [16, 33, 34]. On the other hand, PI_abs_ and F_v_/F_m_ data do not support the view that UV-C light exerts negative effects on lettuce and grapevine plants at the doses considered in this study, notably 1 s treatments, since both parameters were increased compared to that of the control.

### Flashes of UV-C light can be at the origin of systemic effects

Clearly, the systemic effect of flashes of UV-C light we observed (Fig 6) and the efficacy against several pathogens are consistent with what we know about systemic acquired resistance (SAR). Systemic immune responses, notably SAR, can be activated in plants in response to pathogen infection [35–37], and they typically confer broad and long lasting resistance at the whole plant level, even against unrelated pathogens [35, 36, 38].

Several chemical inducers have been identified as being directly or indirectly involved in SAR, including salicylic acid and methyl salicylate [39, 40], jasmonic acid [41], azelaic acid [42], auxin [43], glycerol-3-phosphate [44], pipecolic acid [45], and dehydroabietinal [46]. So far the focus has mainly been on salicylic acid and jasmonic acid, which are still believed to be the key-players in SAR. The importance of their role is regularly confirmed even as our view of plant immunity evolves. For instance, it has been found that salicylic acid and jasmonic acid influence epigenetic responses [47], whereas there is also evidence for epigenetic control of the salicylic acid and the jasmonic acid pathways [48]. There have been many attempts to induce SAR by applications of chemical, biological or, more rarely, physical treatments. SAR can notably be induced by exogenous applications of benzo-(1,2,3)-thiadiazole-7-carbothioic acid (BTH), an analogue of salicylic acid [49, 50]. There are not many studies about the effects of UV-C light on SAR but UV-C light stimulated salicylic acid accumulation in tobacco leaves [51]. Therefore, future studies should test whether the stimulating effect on plant defences of flashes of UV-C light involves the salicylic acid pathway.

From a practical point of view, assessing the systemic effects of UV-C light treatments is needed to define the efficient size and position of lamps for crop treatments. Similarly, there is the need to investigate the role of repetition of UV-C light treatments over time, keeping in mind that the running cost of frequent crop treatments is probably not economically acceptable for farmers and growers. If salicylic acid and SAR are proven to be key players in the immunity conferred by flashes of UV-C light to plants, long-lasting effects are to be expected [38], which could represent an incentive for investigating the possibility of increasing the time between treatments.

## Conclusions

Our observations clearly show that flashes of UV-C light of 1 s have the potential to drive plant defences, probably over a large range of crops and pathogens, opening the way for field and greenhouse treatments. Moreover, our observations show that flashes have the additional and unexpected effect of being more efficient than prolonged irradiations. We found evidence for systemic effects in the four species studied. At this stage it seems very tempting to treat crops with flashes of UV-C light. To develop flashes of UV-C light as a technique for field treatments, more studies are needed to better characterize systemic effects. The duration of resistance will also need to be assessed since it will determine the frequency of treatments. More fundamental research should be performed to unravel the mechanisms of perception of UV-C flashes and the signalling and metabolic pathways triggered or stimulated by them. It is indeed surprising that UV-C flashes of one second are more effective than 60 s irradiation, hinting at the existence of specific physiological responses.

## Supporting information

S1 Data (XLSX)

S2 Data (XLSX)

S3 Data (XLSX)

S4 Data (XLSX)

S5 Data (XLSX)

S6 Data (XLSX)

S7 Data (XLSX)

S8 Data (XLSX)
